# Decoding thalassemia and sickle cell disease: advances in molecular technologies for comprehensive variant detection

**DOI:** 10.3389/fgene.2026.1810737

**Published:** 2026-04-23

**Authors:** Emelie Foord, Darius Sairafi, Monika Berg, Sofia Westerling, Toheeb Adigun, Mehmet Uzunel, Thessalia Papasavva, Michael Uhlin

**Affiliations:** 1 Department of Medicine, Karolinska Institutet, Huddinge, Sweden; 2 Devyser AB, Stockholm, Sweden; 3 Blood Disorder Genetics and Thalassaemia, The Cyprus Institute of Neurology and Genetics (CING), Nicosia, Cyprus

**Keywords:** hemoglobinopathies, molecular diagnostics, next-generation sequencing (NGS), screening follow-up, sickle cell disease, thalassemia

## Abstract

Thalassemia and sickle cell disease are inherited hemoglobinopathies caused by pathogenic variants in the globin genes and represent a major global health burden. Despite major advances in screening and diagnostics, challenges persist due to extensive genetic heterogeneity and complex genotype-phenotype relationships. Conventional workflows typically combine hematologic and biochemical analyses with targeted DNA-based testing. However, traditional molecular approaches are often sequential and labor-intensive, with limited capacity to detect the full spectrum of pathogenic variation. Advances in next-generation sequencing (NGS) now enables integrated and comprehensive strategies to support hemoglobinopathy diagnostics and screening follow-up. Currently available NGS-based platforms allow simultaneous detection of diverse variant classes, including sequence variants and copy number alterations, across multiple disease-relevant genes, including genetic modifiers that may influence disease severity. This review summarizes the genetic basis of thalassemia and sickle cell disease and compiles traditional and emerging molecular testing methodologies. It further discusses the strengths, limitations and utility of NGS-based platforms, and considers their role in shaping future screening and diagnostic workflows for hemoglobinopathies.

## 1 Introduction

Thalassemia and sickle cell disease are autosomal recessive hemoglobinopathies characterized by abnormal hemoglobin production or structure and are among the most prevalent monogenic disorders worldwide. Historically, these conditions have been most common in the Mediterranean region, the Middle East, Southeast Asia and sub-Saharan Africa; however, population migration has led to a broader global distribution, now including regions that previously exhibited low prevalence such as Western Europe and North America (Vichinsky et al., 2005; Kattamis et al., 2020; Musallam et al., 2023).

Together, hemoglobin disorders constitute a substantial global health burden. The World Health Organization (WHO) recognizes hemoglobin disorders as a major public health concern in 70% of countries worldwide, with an estimated 330 000 affected infants born annually (>80% attributable to sickle cell disorders) (Modell and Darlison, 2008). Although global estimates vary considerably and remain subject to large uncertainty due to limitations in diagnostic infrastructure and data collection, persistently high carrier frequencies, incidence rates and associated childhood mortality underscore the impact of these conditions (Modell and Darlison, 2008; Tuo et al., 2024; Piel et al., 2013; Thomson et al., 2023; Manu Pereira et al., 2023; WHO, 2021; Therrell et al., 2015; Zhou et al., 2021; Eleftheriou and Angastiniotis, 2021).

Both thalassemia and sickle cell disease share a common genetic origin in pathogenic variation affecting globin genes; however, distinct molecular mechanisms drive their pathogenesis and clinical manifestations. Diagnostic strategies have traditionally relied on hematologic parameters and biochemical hemoglobin analysis, which remain essential but are inherently limited in their ability to resolve underlying genetic complexity, identify compound or atypical genotypes and support accurate clinical risk assessment. Genetic testing is therefore central to diagnosis, refining disease classification and supporting genetic counseling. However, conventional molecular approaches often rely on sequential, assay-specific testing strategies, each optimized for particular variant types or predefined variant panels, thereby limiting the efficiency and breadth of variant detection.

In this context, next-generation sequencing (NGS) technologies have the potential to improve hemoglobinopathy workflows by enabling integrated comprehensive detection of sequence variants and copy number alterations across multiple genes within a single platform, also facilitating the simultaneous assessment of genetic modifiers known to influence disease severity and clinical course.

This narrative review summarizes current state-of-the-art practices in thalassemia and sickle cell disease, with a focus on available molecular testing methodologies and particular emphasis on emerging NGS-based platforms and their potential to transform future screening and diagnostic workflows. Relevant literature was identified through PubMed and selected for its relevance on the topics covered.

## 2 Genetic regulation of hemoglobin

Hemoglobin (Hb) is the main oxygen-carrying component of erythrocytes and is a tetramer composed of two α-like (α or ζ) and two β-like (β, δ, γ or ε) globin chains, each containing an iron-containing heme group capable of binding oxygen (WHO, 2021). Globin chain synthesis is regulated by two gene clusters: the α-globin gene cluster on chromosome 16 (including the highly homologous *HBA1* and *HBA2* genes and the embryonically expressed *HBZ*) and the β-globin gene cluster on chromosome 11 (including the adult-expressed *HBB* and *HBD*, the embryonically expressed *HBE1* and the fetal-expressed *HBG1* and *HBG2*) (Musallam et al., 2024; Wakabayashi et al., 2022).

**FIGURE 1:**
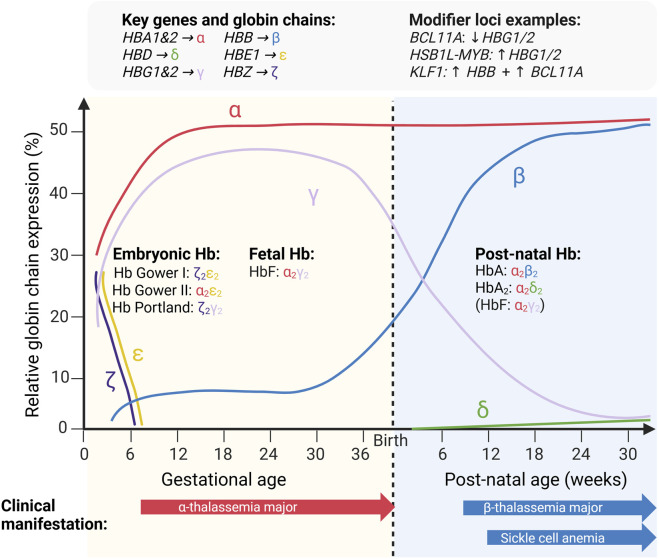
Globin chain expression with associated hemoglobins, encoding genes, examples of modifier loci influencing γ/β-globin regulation and the clinical manifestation of severe hemoglobinopathies during early development and infancy.

During embryonic and fetal development, hemoglobin expression is regulated by a tightly controlled globin gene-switching process involving coordinated activation and repression of the globin genes (Figure 1). This process culminates postnatally in a transition characterized by declining γ-globin and increasing β-globin expression. This final switch drives the replacement of fetal hemoglobin (HbF comprising of two α chains and two γ chains; α_2_γ_2_) with adult hemoglobin (HbA, comprising of two α and two β chains; α_2_β_2_). Within the first year of life, this results in the predominant HbA (95%–98% of the total hemoglobin), accompanied by approximately 2%–3.3% HbA2 (comprising of two α and two δ chains; α2δ2) and less than 1% HbF in healthy individuals (Musallam et al., 2024; Kountouris et al., 2016; Traeger-Synodinos et al., 2015).

## 3 Genetic and clinical basis of thalassemia and sickle cell disease

Thalassemia is caused by reduced or absent synthesis of α-globin chains (α-thalassemia) or β-globin chains (β-thalassemia), typically denoted as α^+^/α^0^ or β^+^/β^0^ respectively. These defects are caused by pathogenic variants in *HBA1* and *HBA2* in α-thalassemia, or in *HBB* in β-thalassemia, leading to imbalanced globin chain synthesis. This disrupts normal hemoglobin assembly and reduces the production of functional hemoglobin. The resulting excess of unpaired globin chains forms unstable, non-functional globin complexes, representing a central pathogenic mechanism in thalassemia. Other, less common forms of thalassemia exist but are not discussed further in this review.

In contrast, sickle cell disease comprises a group of structural hemoglobinopathies caused by pathogenic variants in *HBB* that result in the production of hemoglobin S (HbS), a structurally altered hemoglobin variant. Homozygous inheritance of HbS, known as sickle cell anemia, is the most common and severe form of the disease. Additional disease-associated genotypes arise from compound heterozygosity involving co-inheritance of HbS and other abnormal β-globin variants (Zhou et al., 2021; Kirkham et al., 2023). Sickle cell trait refers to individuals heterozygous for the HbS variant in the presence of a normal HbA allele (HbAS). The high prevalence of the HbS allele in certain populations is thought to reflect the selective pressure described by the malaria hypothesis, which suggests that individuals with the heterozygous carrier state have a protective advantage against malaria (Kirkham et al., 2023; Piel et al., 2010).

Over the past decades, advances in molecular techniques have greatly expanded the number of known variants and their association with thalassemia and sickle cell disease. Additionally, the global variant databases, such as ClinVar, HbVar and IthaNet, have become important tools facilitating knowledge sharing, variant reporting and interpretation. To date, the HbVar database catalogs more than 1900 hemoglobin-related sequence variants, including more than 500 thalassemia-associated variants (HbVar, 2026).

### 3.1 α-Thalassemia

α-thalassemia results from pathogenic variants, most often large deletions (copy number variants, CNVs), affecting the *HBA1* and *HBA2* genes (Kattamis et al., 2022). Common large deletions spanning both α-globin genes include the Southeast Asian deletion (--SEA), the Filipino deletion (--FIL) and the Mediterranean deletion (--MEDI and MEDII), whereas common deletions affecting a single α-globin gene include the 3.7-kb deletion (-α3.7) and the 4.2-kb deletion (-α4.2) (Musallam et al., 2024; Tamary et al., 2024). Non-deletional sequence variants causing α-thalassemia are less common and include, for example, Hb Constant spring, caused by a pathogenic single nucleotide variant (SNV) that results in an unstable mRNA transcript and abnormally elongated α-globin chains (Musallam et al., 2024; Tesio and Bauer, 2023).

Clinical severity is determined primarily by the number of functional α-globin genes, which also forms the basis of the traditional disease subtypes outlined in Table 1 (Harteveld et al., 2010). However, there is considerable clinical heterogeneity reflected not only by α-globin gene dosage but also by the specific genes involved, *cis* or *trans* configuration, alterations in regulatory regions, compound heterozygosity and additional modifying factors that influence phenotypic severity (Tamary et al., 2024; Vichinsky, 2010). Consequently, individuals with α-thalassemia may present with a broad spectrum of clinical manifestations.

**TABLE 1:** Disease subtypes in thalassemia and their associated genotypes and clinical features.

Disease subtype	Genotype	Clinical features
α-thalassemia		
α-thalassemia minima (silent carrier)	One defective or deleted α-globin gene (-α/αα)	Clinically asymptomatic; carrier status with reproductive risk
α-thalassemia minor (trait)	Two defective or deleted α-globin genes (-α/-α or --/αα)	Usually asymptomatic; may present with mild to moderate microcytic hypochromic anemia
α-thalassemia intermedia (HbH disease)	Three defective or deleted α-globin genes (−/−α, various genotypes)	Excess β-globin chains form unstable β_4_ tetramers (HbH) causing moderate to severe hemolytic anemia
α-thalassemia major (Hb Bart’s hydrops fetalis syndrome)	Four defective or deleted α-globin genes (−/−, various genotypes)	Excess γ-globin chains form non-functional γ_4_ tetramers (Hb Bart’s), leading to severe impairment of fetal development. Fatal *in utero* or shortly after birth
β-thalassemia		
β-thalassemia minor (trait)	Heterozygosity for a pathogenic *HBB* variant, resulting in reduced (β^+^) or absent (β^0^) β-globin production from the affected allele	Usually asymptomatic or mildly symptomatic; mild microcytic hypochromic anemia
β-thalassemia intermedia	Homozygous or compound heterozygous *HBB* variants with residual β-globin production	Variable anemia (mild to moderate); transfusions not required or only occasionally needed
β-thalassemia major (Cooley’s anemia)	Homozygous or compound heterozygous *HBB* variants (often β^0^/β^0^ or β^0^/β^+^) with absent or minimal β-globin production	Severe anemia with early-onset transfusion dependency

The most severe form of α-thalassemia, Hb Bart’s hydrops fetalis, manifests during fetal development. Non-functional homotetramers of γ chains (γ_4,_ referred to as Hb Bart’s) predominate, while embryonic Hb Portland (ζ_2_γ_2_) may provide oxygen transport and enable survival into the second or third trimester (Figure 1) (Tesio and Bauer, 2023). Clinical features include cardiac failure, hepatosplenomegaly, impaired brain growth, skeletal deformations, placental enlargement and fatal outcome *in utero* or shortly after birth (Harteveld et al., 2010). In the intermediate to severe form of α-thalassemia, called hemoglobin H (HbH) disease, less than 30% of the normal amount of α-globin is produced. The predominant clinical feature is anemia with variable levels of HbH (0.8%–40%), comprised of unstable homotetramers of β chains, (β_4_) and sometimes Hb Bart’s in the peripheral blood. Other clinical signs include splenomegaly, jaundice and growth retardation in children. Patients can also experience acute hemolytic episodes in response to drugs and infections, leg ulcers, gallstones and more (Harteveld et al., 2010).

### 3.2 β-thalassemia

β-thalassemia arises from pathogenic variants in *HBB,* most commonly SNVs, that reduce (β^+^) or abolish (β^0^) β-globin chain synthesis, resulting in an accumulation of excess unpaired α-globin chains, ineffective erythropoiesis and hemolysis. Altogether, this imbalance underlies a wide range of clinical manifestations and disease subtypes as outlined in Table 1 (Taher et al., 2021). Although there is considerable genetic heterogeneity, certain sequence variants are particularly common in specific geographical regions. For example, [*HBB*:c.93-21G>A] and [*HBB*:c.118C>T] (historically referred to as IVS I-110 G>A and CD 39 CAG>TAG) are prevalent in the Mediterranean and Middle East, respectively, [*HBB*:c.126_129delCTTT] is common in Southeast Asia and [*HBB*:c.92 + 5G>C] is frequent in South Asia (also known as (CD 41/42 (-TTCT) and IVS I-5 (G>C), respectively) (Tesio and Bauer, 2023). The prevalence of different sequence variants is highly dependent on the region and the population. To further understand the molecular spectrum, it is recommended to assess region-specific studies, such as, for example, by Kountouris et al. for Cyprus (Kountouris et al., 2016). As exemplified in this study, even within a small country, there may be considerable heterogeneity across different areas (Kountouris et al., 2016).

Clinical severity in β-thalassemia is modulated by the specific *HBB* variants, co-inheritance of α-thalassemia and other genetic modifiers influencing globin balance or HbF expression to compensate for the lack of β-globin chains (Traivaree et al., 2018). Similar to α-thalassemia, the genotype and resulting phenotype of the disease are highly complex. Clinically, untreated severe disease leads to growth failure, hepatosplenomegaly and skeletal changes due to marrow expansion and extramedullary hematopoiesis. The most severe form of β-thalassemia, the major subtype (sometimes referred to as Cooley’s anemia), usually manifests within the first 2 years of life and requires regular lifelong blood transfusions. Babies born with two defective β-globin genes are usually healthy at birth; however, the disease typically begins to manifest around 6 months of age or earlier, when HbF declines and is supposed to be replaced by HbA as part of the final hemoglobin switching process (Figure 1) (WHO, 2021). The Intermedia subtype usually presents later in life. Additionally, there are forms of β-thalassemia associated with hemoglobin anomalies, including HbC/β-thalassemia and HbE/β-thalassemia, both of which are associated with a wide variability in clinical phenotype and severity (Galanello and Origa, 2010).

### 3.3 Transfusion dependency as a clinical classification for thalassemias

Clinically, thalassemias are commonly classified as non-transfusion-dependent thalassemia (NTDT) or transfusion-dependent thalassemia (TDT), a distinction that guides patient management strategies. Transfusion dependency necessitates regular blood transfusions and proactive prevention of transfusion-related complications, most notably iron overload, as the human body lacks an effective mechanism for iron excretion. Consequently, iron chelation therapy is essential for preventing iron-related organ toxicity and associated morbidity (Galanello and Origa, 2010). In contrast, the management of NTDT focuses on monitoring and treating complications arising from chronic anemia and ineffective erythropoiesis (Musallam et al., 2024). Although survival outcomes have improved in high-income settings, life expectancy remains reduced, particularly in low- and middle-income countries and among patients with transfusion-dependent thalassemia (Kattamis et al., 2022).

### 3.4 Sickle cell disease

Sickle cell disease is caused by pathogenic variants in *HBB* and includes several distinct genotypes. Sickle cell anemia results from homozygosity for hemoglobin S (HbSS), while other compound heterozygous forms include HbSC disease (HbSC) and Sickle cell β-thalassemia (including HbS/β^+^-thalassemia and HbS/β^0^-thalassemia), in which HbS is co-inherited with another pathogenic β-globin variant.

At the molecular level, HbS results from a single SNV in *HBB* (c.20A>T), causing an amino acid substitution (Glu→Val) in the β-globin chain, which introduces hydrophobic interactions and promotes polymerization of deoxygenated hemoglobin. HbC results from another distinct SNV in *HBB* (c.19G>A), leading to another amino acid substitution (Glu→Lys), which alters the charge properties and promotes hemoglobin crystallization. In sickle β-thalassemia, the molecular impact of the specific co-inherited β-thalassemia variant on β-globin synthesis determines the residual hemoglobin composition and drives disease severity (Rees et al., 2010; Pecker and Lanzkron, 2021).

Under deoxygenated conditions, polymerization of HbS leads to erythrocyte deformation into the characteristic sickle shape, increased rigidity and adhesiveness, hemolysis and vaso-occlusion through microvascular obstruction. These processes underlie the hallmark clinical complications of sickle cell disease, including recurrent painful vaso-occlusive crises, acute chest syndrome, stroke, increased susceptibility to infections and progressive multi-organ damage (Zhou et al., 2021; Pecker and Lanzkron, 2021; MacLean et al., 2008). As in thalassemia, clinical severity in sickle cell disease varies widely, particularly among compound heterozygous genotypes, and is influenced by residual β-globin synthesis, genetic modifiers affecting HbF levels, co-inheritance of α-thalassemia and environmental factors (Kirkham et al., 2023; Kavanagh et al., 2022; Rumaney et al., 2014). The most severe form, sickle cell anemia, typically begins to manifest in early infancy as HbF levels decline and HbS becomes the predominant hemoglobin, enabling polymerization of deoxygenated HbS and subsequent red blood cell sickling (Figure 1). Individuals with sickle cell trait are typically asymptomatic, as they generally have HbS concentrations of 40% or less of the total hemoglobin level. With normal HbA as the dominant form, the erythrocytes usually do not deform and blood counts are typically normal. However, these individuals have an increased risk of developing kidney disease, as sickling of erythrocytes can occur in the renal medulla (Pecker and Lanzkron, 2021). Despite advances in management, sickle cell disease remains associated with substantially reduced life expectancy worldwide, with particularly high childhood mortality in resource-limited settings (Kavanagh et al., 2022; Jiao et al., 2023).

## 4 Genetic modifiers for β-thalassemia and sickle cell disease

A major determinant of disease severity in β-thalassemia and sickle cell disease is the persistence or reactivation of HbF expression, which is associated with reduced morbidity and mortality (Wakabayashi et al., 2022). This has driven extensive research into the regulatory mechanisms governing the hemoglobin switching process, with genome-wide association studies (GWAS) playing a central role in identifying genetic modifiers of HbF production and their relevance to phenotype prediction and therapeutic targeting. In contrast, increased γ-globin expression does not compensate for α-thalassemia, as α-globin is required for the formation of functional HbF (α_2_γ_2_), rendering HbF-based modulation clinically irrelevant in the context of α-thalassemia.

Several well-characterized genetic modifiers and regulatory sequence variants have been shown to influence HbF levels and clinical severity in β-globin-related hemoglobinopathies, including variants in *BCL11A*, the *HBS1L-MYB* intergenic region, *KLF1* and loci within *HBD* and *HBG1/HBG2*. *BCL11A*, located on chromosome 2, encodes a transcription factor that acts as a major repressor of γ-globin expression (Tesio and Bauer, 2023). Variants such as rs11886868 (Uda et al., 2008) and rs1427407 (Danjou et al., 2012) are strongly associated with elevated HbF levels and milder clinical phenotypes in both β-thalassemia and sickle cell disease, partially alleviating the globin chain imbalance (Wakabayashi et al., 2022; Rumaney et al., 2014; Uda et al., 2008; Danjou et al., 2012; Ojewunmi et al., 2019; Danjou et al., 2015). Notably, therapeutic downregulation of *BCL11A* is now being utilized in gene therapy strategies aimed at enhancing HbF production in β-thalassemia and sickle cell disease (EMA, 2023; Esric et al., 2021; Frangoul et al., 2024).

Variants within the *HBS1L-MYB* intergenic region on chromosome 6 represent another class of HbF modifiers, with multiple SNPs, including, for example, rs9399137, associated with increased HbF levels and milder disease courses (Kirkham et al., 2023; Tesio and Bauer, 2023; Uda et al., 2008; Danjou et al., 2012; Menzel et al., 2007). The contribution of these polymorphisms to interindividual HbF variability has been confirmed in numerous studies (Rumaney et al., 2014; Ojewunmi et al., 2019; Danjou et al., 2015). For example, Danjou et al. demonstrated that HbF-associated variants in *HBS1L-MYB*, together with *BCL11A* polymorphisms and α-globin gene defects, significantly influenced β-thalassemia severity, as assessed by the age at first transfusion (Danjou et al., 2012).

The transcription factor Kruppel-like factor 1 (KLF1), encoded by the *KLF1* gene located on chromosome 19, plays a central role in erythroid differentiation and globin gene regulation. KLF1 promotes adult β-globin expression through its interaction with the β-globin promoter and indirectly suppresses HbF through the activation of *BCL11A* (Tesio and Bauer, 2023). Variants in *KLF1*, as well as in the γ-globin-encoding genes *HBG1/HBG2*, further contribute to HbF variation and clinical heterogeneity in β-thalassemia and sickle cell disease (Rumaney et al., 2014; Danjou et al., 2015; Yin et al., 2022-9). Consistent with these findings, a recent large systematic review and meta-analysis by Kirkham et al. consolidated current knowledge on genetic modifiers in sickle cell disease, identifying 19 SNVs across *BCL11A*, *HBS1L-MYB* and *HBG2* that were significantly associated with HbF levels (Kirkham et al., 2023). These findings highlight the central role of HbF-modulating loci in shaping disease onset and phenotype.

An increased understanding and clinical translation of genetic modifiers are key to advancing risk stratification and support individualized management of β-globin-related hemoglobinopathies. Ongoing efforts to integrate modifier information, including the development of composite genetic scores, aim to improve prediction of disease severity and facilitate clinical implementation (Danjou et al., 2015). However, the routine use of modifier testing in clinical practice remains variable and is not yet universally adopted. While modifier information may contribute to improved phenotypic prediction in affected individuals, the predictive performance of composite genetic scores may vary across populations, reflecting differences in genetic background and modifier allele frequencies, highlighting the need for continued refinement.

Moreover, interpretation and counseling challenges arise in screening settings. As clinical relevance is context-dependent, modifier variants identified incidentally through comprehensive assays often have limited or uncertain predictive value. Cautious interpretation is therefore essential to avoid over-extrapolating prognostic meaning in situations where HbF-modifying variants have limited or no clinical relevance.

In summary, HbF modifying loci are central to understanding clinical variability, disease severity and adapting management in individuals affected by β-globin-related disorders. This highlights the potential clinical value of integrating modifier analysis into the genetic evaluation, while emphasizing the importance of interpreting such findings within appropriate clinical contexts. Additional genetic modifiers beyond the ones mentioned may also influence disease severity, as reviewed further by, *e.g.*, Tesio and Bauer for thalassemia (Tesio and Bauer, 2023) and Tsukahara et al. for sickle cell disease (Tsukahara et al., 2024).

## 5 Screening strategies for hemoglobinopathies

### 5.1 Newborn screening

In recent decades, newborn screening (NBS) programs for hemoglobinopathies have been implemented in many high-prevalence and ethnically diverse regions, including Europe and North America (Manu Pereira et al., 2023; Daniel et al., 2019) (Zhou et al., 2021). In the US, universal NBS for sickle cell trait and disease has been implemented in all states since 2006, with New York State being the first pilot in 1975 (Pecker and Lanzkron, 2021; Center for Disease control and Prevention, 2015; Quarmyne et al., 2025). The primary objective of NBS is to enable early diagnosis and timely initiation of care, leading to improved survival and quality of life for affected individuals and their families (Zhou et al., 2021; Center for Disease control and Prevention, 2015; Bain et al., 2023). For example, early identification of thalassemia enables close monitoring of transfusion needs, whereas in sickle cell disease, early initiation of penicillin prophylaxis substantially reduces the risk of life-threatening infections (Bain et al., 2023).

In countries with established screening infrastructure, first-line NBS strategies typically include high-performance liquid chromatography (HPLC), capillary electrophoresis-based techniques, and, in some settings, tandem mass spectrometry, using dried blood spots as the primary specimen type (Bain et al., 2023). Screen-positive results are subsequently confirmed using secondary biochemical or molecular methods, as the definitive identification of hemoglobin variants requires protein- or DNA-based analysis (Center for Disease control and Prevention, 2015; Bain et al., 2023). Screening algorithms may vary according to disease type, geographic region and laboratory setting (Center for Disease control and Prevention, 2015). Despite the demonstrated benefits, universal NBS remains unavailable or limited in many high-prevalence, resource-limited settings, highlighting persistent global disparities in early diagnosis (Ojewunmi et al., 2019; World Health Organization, 2020).

### 5.2 Carrier and premarital/preconception screening

The WHO has estimated that approximately 1.1% of couples worldwide are considered at risk of having children with a hemoglobin disorder (Modell and Darlison, 2008). Carrier screening aims to identify asymptomatic individuals with hemoglobinopathy traits in order to support genetic counselling and informed reproductive decision-making for at-risk couples. In the context of sickle cell trait, carrier identification may also increase awareness of associated health risks (Pecker and Lanzkron, 2021). Premarital or preconception screening programs, which are mandatory in some countries (*e.g.*, Cyprus, Saudi Arabia, Kuwait, and Turkey (Al-Shroby et al., 2021; Rouh et al., 2021; Ithanet, 2026)), have proven effective in reducing births affected by severe hemoglobinopathies through early risk assessment and counselling (Modell and Darlison, 2008; WHO, 2021; Kountouris et al., 2016; Wang et al., 2023). Long-standing programs, such as those in Greece and Cyprus (Kountouris et al., 2016; Turner et al., 2016), as well as more recent initiatives in China (Wang et al., 2023), have led to marked reductions in the incidence of severe thalassemia (WHO, 2021; Taher et al., 2021; Al-Shroby et al., 2021; Rouh et al., 2021; Wang et al., 2023; Turner et al., 2016). However, challenges persist, including limited resources in some regions and variable public awareness (Wang et al., 2023).

Reflecting increasing population diversity, screening guidelines have evolved toward more universal approaches. The American College of Obstetricians and Gynecologists (ACOG) reports that approximately 1 in 66 people in the US carry hemoglobinopathy trait. The ACOG now recommends offering universal hemoglobinopathy testing to individuals planning pregnancy or at the initial prenatal visit, replacing earlier ancestry-based strategies (ACOG Committee on Obstetrics, 2007; ACOG, 2022). Recommended testing approaches include hemoglobin electrophoresis or molecular genetic testing, with exemplified expanded carrier screening including sickle cell disease and other hemoglobinopathies (ACOG, 2022). Effective carrier and premarital screening programs rely on accurate, reliable, and preferably high-throughput laboratory methods, and a range of testing strategies has been developed to meet these requirements (Turner et al., 2016).

## 6 Diagnostic strategies

Clinical practice in hemoglobinopathy diagnostics is guided by recommendations from organizations such as the Thalassemia International Federation (TIF) (Farmakis et al., 2021) and the European Molecular Quality Network (EMQN) Best Practice Guidelines for Hemoglobinopathies (Traeger-Synodinos et al., 2015), alongside national guidelines (*e.g.*, (Bain et al., 2023)) and disease-specific consensus documents.

### 6.1 Prenatal and pre-implantation genetic diagnosis

Prenatal diagnosis may be performed by molecular analysis of fetal DNA obtained from chorionic villus sampling or amniotic fluid to detect disease-causing variants. However, invasive fetal sampling is associated with procedural risks, underscoring the importance of preconception screening when feasible. Non-invasive prenatal diagnosis by analyzing cell-free fetal DNA in maternal plasma is considered experimental and is not currently recommended by the ACOG (ACOG, 2022).

In settings with access to advanced reproductive technologies, pre-implantation genetic diagnosis enables selection of embryos without disease-causing variants during *in vitro* fertilization. This approach may offer at-risk couples a reproductive option to reduce the likelihood of having children affected by severe hemoglobinopathies. Further descriptions of strategies for prenatal and pre-implantation diagnostics are beyond the scope of this review.

### 6.2 Diagnostic workflows for thalassemia

**FIGURE 2:**
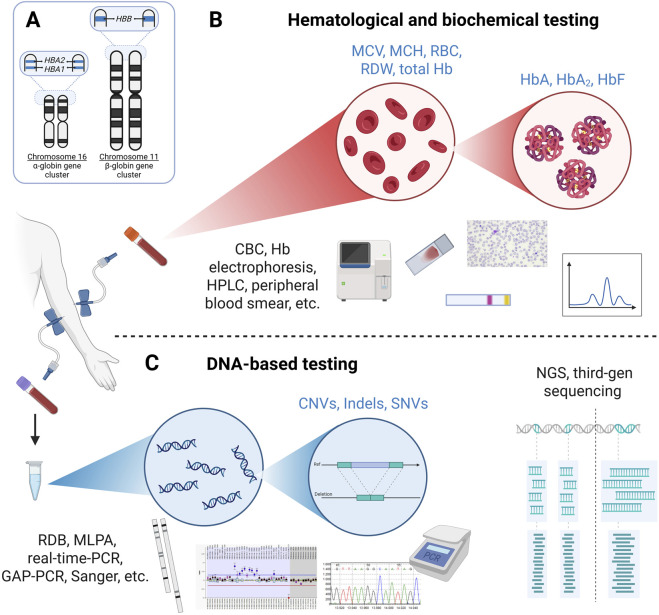
Overview of the diagnostic laboratory workflow for hemoglobinopathies. **(A)** Schematic overview of the α- and β-globin gene clusters, including four α-globin and two β-globin gene copies in a healthy individual. **(B)** Hematological and biochemical testing for suspected thalassemia and sickle cell disease typically includes complete blood count (CBC) parameters, hemoglobin (Hb) electrophoresis, high-performance liquid chromatography (HPLC) and peripheral blood smear analysis. **(C)** DNA-based testing strategies range from traditional approaches, such as reverse dot blot (RDB) hybridization, GAP-PCR, multiplex ligation-dependent probe amplification (MLPA), real-time PCR and Sanger sequencing, to more comprehensive methods such as next-generation sequencing (NGS) and third-generation (long-read) sequencing for detection of pathogenic variants, including copy number variants (CNVs), insertion/deletions (indels) and single nucleotide variants (SNVs). The order and selection of testing strategies may vary depending on the suspected disorder, variant type and regional/laboratory practice. Abbreviations: MCV, mean corpuscular volume; MCH, mean corpuscular hemoglobin; RBC, red blood cell count; RDW, red cell distribution width.

The diagnosis of thalassemia integrates clinical presentation with laboratory findings while acknowledging the inherent limitations of the hematological, protein-based and molecular analyses used (Bain et al., 2023). The initial evaluation typically includes a complete blood count (CBC) with key red blood cell parameters, such as mean corpuscular volume (MCV) and mean corpuscular hemoglobin (MCH), which are measurements of red blood cell size and hemoglobin content, respectively (Figure 2). Additional parameters, such as red blood cell (RBC) count, Hb concentration and red cell distribution width (RDW), are often included in the evaluation (Traeger-Synodinos et al., 2015; Kattamis et al., 2022). In adults, MCV <79 fL and MCH <27 pg are commonly used screening cut-offs, with values below these thresholds warranting continued investigation for thalassemia (Traeger-Synodinos et al., 2015).

HPLC or capillary electrophoresis are commonly used methods to analyze hemoglobin patterns, detect hemoglobin variants and quantify hemoglobin fractions, such as HbA_2_ and HbF. Elevated HbA_2_ and HbF levels are often observed as compensatory responses in individuals with β-thalassemia and other hemoglobinopathies and are therefore useful in the diagnostic workflow (Traeger-Synodinos et al., 2015; Kattamis et al., 2022). In adults, HbA_2_ levels >3.5% of total hemoglobin are suggestive of heterozygosity for β-thalassemia, while an HbF level >2% warrants further investigation (Traeger-Synodinos et al., 2015; Kattamis et al., 2022).

Additional hematological, protein-based and molecular analyses may be employed to follow-up or confirm abnormal or ambiguous findings from initial tests. These may include assessment of red cell morphology, globin chain synthesis or advanced characterization of hemoglobin variants using mass spectrometry, isoelectric focusing and/or DNA-based methods (Traeger-Synodinos et al., 2015). Given the complexity of thalassemia genetics and genotype-phenotype correlations, diagnosis of thalassemia patients and carrier status can still remain challenging (Munkongdee et al., 2020). Molecular testing has therefore become increasingly essential within the diagnostic and screening follow-up workflow, particularly in cases where conventional analyses are inconclusive or yield borderline results, enabling definitive variant identification and resolution of genetically complex cases (Kattamis et al., 2022).

### 6.3 Diagnostic workflows for sickle cell disease

Evaluation of suspected sickle cell disease typically includes complete blood count, reticulocyte count (reflecting the level of immature erythrocytes), peripheral blood smear (for assessment of the characteristic sickle-shaped cells and other morphological abnormalities) and hemoglobin electrophoresis. Hemoglobin electrophoresis determines the relative concentration of different hemoglobin fractions, helping to identify abnormalities, confirming diagnosis, and, if relevant, determining the disease subtype (Pecker and Lanzkron, 2021; Kavanagh et al., 2022). However, traditional hematologic and biochemical methods may not reliably differentiate Sβ^0^-thalassemia from sickle cell anemia (HbSS), highlighting the utility of *HBB* gene sequencing (de Martino et al., 2019). Also, the use of the outlined traditional hematology-based analyses is limited in defining carrier states and compound heterozygote genotypes (Adekunle et al., 2021).

## 7 Molecular technologies for variant detection

### 7.1 Traditional molecular approaches

Hemoglobinopathies are unique among genetic disorders in that carrier detection has historically relied primarily on hematologic and biochemical assays rather than DNA-based analysis, as noted by the EMQN Best Practice Guidelines (Traeger-Synodinos et al., 2015). Nevertheless, molecular testing has become increasingly important in both screening follow-up and diagnostic workflows, particularly to improve diagnostic accuracy and resolve complex or atypical genotypes (Traeger-Synodinos et al., 2015; Bain et al., 2023; Farmakis et al., 2021). In routine clinical practice, hemoglobin variants identified at initial screening are confirmed using an independent secondary or alternative method (Center for Disease control and Prevention, 2015; Bain et al., 2023).

**FIGURE 3:**
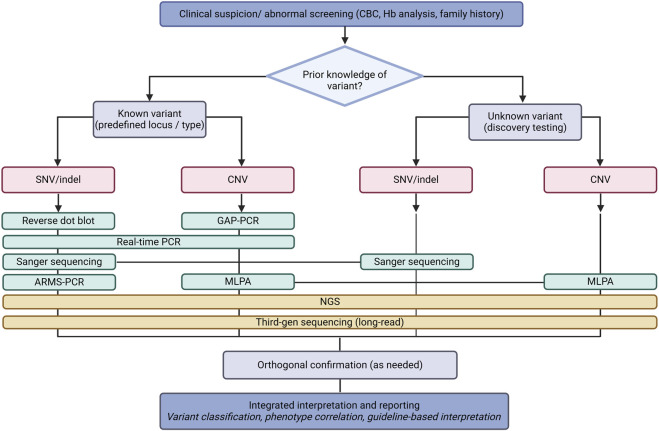
Schematic of molecular testing workflows for hemoglobinopathies. Flowchart of DNA-based testing approaches following clinical suspicion or abnormal screening results. Traditional targeted assays are typically applied in a stepwise manner based on prior knowledge and the expected variant type, while next-generation sequencing (NGS) and third-generation sequencing provide comprehensive approaches suitable for both known and unknown variants. Although the figure separates “known” and “unknown” variant pathways for conceptual clarity, in practice NGS may be used as first-line or follow-up testing regardless of prior variant knowledge. Testing strategies may also vary according to, *e.g.*, local laboratory workflows, infrastructure availability and regional recommendations.

A range of DNA-based techniques are currently used in routine practice (Table 2) (Figure 2). Traditional molecular methods include GAP-PCR, amplification refractory mutation system (ARMS)-PCR, real-time PCR, reverse dot blot hybridization, multiple ligation-dependent probe amplification (MLPA) and Sanger sequencing (Traeger-Synodinos et al., 2015; Quarmyne et al., 2025; Munkongdee et al., 2020). Selection of an appropriate method depends on the testing objective and variant type; for example, Sanger sequencing is commonly used to identify unknown SNVs, whereas MLPA is applicable for detecting large deletions or duplications (Figure 3) (Traeger-Synodinos et al., 2015). Practical considerations such as workload, sample type (*e.g.*, blood or dried blood spots), ease of handling, reproducibility, laboratory expertise and cost further influence method selection (Bain et al., 2023). As emphasized by Munkongdee et al., laboratories should adopt the techniques they are most familiar with and that are cost-effective for routine use (Munkongdee et al., 2020). This consideration is especially important in low- and middle-income countries, where demand for genetic testing is high and resources may be limited (Heavenlight et al., 2021).

**TABLE 2:** Overview of molecular methods for genetic variant detection in thalassemia and sickle cell disease.

Method	Variant types	Strengths	Limitations	Commercial assays
Sanger sequencing	SNVs and indels within targeted regions	High base-level accuracy; well established; gold standard for variant confirmation	Labor-intensive and time consuming; low throughput; limited scalability; cannot detect CNVs or structural variants; requires prior target selection	N/A
Multiple ligation-dependent probe amplification (MLPA)	CNVs (deletions and duplications)	Robust detection of known CNVs; cost-effective; widely validated	Cannot detect SNVs or indels; limited breakpoint resolution; probe-dependent; restricted to targeted regions	SALSA MLPA Probemix P140 HBA; SALSA MLPA Probemix P102 HBB (MRC Holland)
GAP-PCR	Known large deletions with defined breakpoints (*e.g.*, common α-thalassemia deletions)	Rapid, inexpensive, highly specific for targeted deletions	Limited to predefined variants; unable to detect novel, complex or atypical rearrangements	N/A
Reverse dot blot (RDB) hybridization	Panels of predefined SNVs and indels	Rapid screening of common variants; simple workflow; useful in high-prevalence regions	Restricted to known variants on the panel; cannot detect CNVs or rare/novel variants; limited flexibility for panel expansion	α-Globin StripAssay*; β-globin StripAssay MED/IME/SEA (3 separate products); β-thal Modifier StripAssay (ViennaLab Diagnostics) Alpha Globin Test; Beta Globin Test; Beta Globin Plus Test (Nuclear Laser Medicine)
Real-time PCR (qPCR)	Targeted SNVs, indels and copy number changes	Quantitative; rapid turnaround; suitable for targeted confirmatory testing	Assay-specific; limited multiplexing; not comprehensive; requires prior knowledge of targets	N/A
Amplification refractory mutation system (ARMS)-PCR	Known SNVs and indels	Simple, rapid, and cost-effective; high specificity for targeted variants	Limited to predefined variants; not scalable; cannot detect CNVs or novel variants; requires careful primer design	N/A
Next-generation sequencing (NGS)	SNVs, indels, CNVs, genetic modifier variants (assay-dependent)	High throughput; simultaneous detection of multiple variant classes; enables integrated analysis of disease genes and modifiers	Higher cost and bioinformatic complexity; CNV and complex structural variant detection may be assay- and coverage-dependent; challenges in homologous regions (*e.g*., *HBA1/HBA2*); variant interpretation burden	Devyser Thalassemia; Devyser Thalassemia v2* (Devyser AB) THALASSEMIA panel (4bases)
Third-generation (long-read) sequencing	SNVs, indels, CNVs, complex structural variants (incl. Rearrangements and gene conversions)	Resolves complex structural variants and homologous regions; improved haplotype phasing and breakpoint resolution	Higher cost; lower throughput; limited clinical validation and routine diagnostic adoption	N/A

* IVDR-certified products. Abbreviations: CNV, copy number variants; SNV, single nucleotide variant.

Routine workflows often rely on pre-defined variant panels, such as targeted testing for common α-thalassemia deletions. When results are inconclusive or require further clarification or confirmation, sequential application of complementary methods is frequently required. Such strategies may be less effective in genetically diverse populations and offer limited flexibility for regional customization or detection of rare and complex genotypes (Turner et al., 2016; Fan et al., 2023; Hamid et al., 2021; Rossarin et al., 2024). Consequently, standard panels may face challenges in efficiency and in distinguishing certain genotypic combinations, motivating the development and implementation of more comprehensive approaches. Figure 3 illustrates various testing pathways based on known and unknown variant scenarios, however real-world diagnostic workflows are often iterative and may combine targeted assays with more comprehensive sequencing approaches depending on clinical context and other factors.

### 7.2 Methods for comprehensive variant detection

The extensive genetic heterogeneity of hemoglobinopathies, combined with the diversity of variant types, multiple disease-relevant genes and the need for high-throughput testing pose challenges for traditional molecular techniques. NGS (Selvatici et al., 2024; Zulkeflee et al., 2023; Zhao et al., 2020) and, more recently, third-generation sequencing (Fan et al., 2023; Peng et al., 2022; Liang et al., 2023) technologies, have emerged as approaches capable of addressing many of these limitations (Peng et al., 2022). NGS can detect multiple variant classes within several globin and modifier loci in a single assay, which is advantageous for the molecular complexity associated with thalassemia and sickle cell disease (Table 2) (Figure 3) (Adekunle et al., 2021). It has the ability to support testing in genetically diverse and admixed populations, enabling the detection of common, population-specific, rare and previously uncharacterized variants (Yin et al., 2022-9; Adekunle et al., 2021; Selvatici et al., 2024; Zulkeflee et al., 2023). NGS can also facilitate characterization of sickle cell disease genotypes, including compound heterozygote forms that are challenging to resolve efficiently using conventional methods (Adekunle et al., 2021).

Despite these advantages, several technical limitations should be considered when applying NGS in hemoglobinopathy workflows. Detection of CNVs using short-read sequencing remains dependent on sequencing depth, panel design and bioinformatic normalization strategies, resulting in platform- and pipeline-dependent performance. In addition, high sequence homology, particularly between *HBA1* and *HBA2*, can complicate read alignment and variant assignment. In addition, short-read approaches may also have limited ability to fully resolve complex structural variants, large rearrangements or repetitive regions within globin loci, which may require complementary methods such as MLPA, GAP-PCR or long-read sequencing for confirmation. By expanding the range of detectable variants, comprehensive sequencing increases the likelihood of detecting variants of uncertain significance (VUS), which introduces additional challenges for clinical interpretation and genetic counselling.

Multiple population-level studies have demonstrated the utility of NGS in hemoglobinopathies. Shang et al. evaluated an NGS-based panel targeting all eight globin genes and key modifier loci (*KLF1, BCL11A, HBS1L* and *MYB)* in a large Chinese cohort including 2522 individuals with hemoglobinopathies and 10111 couples undergoing carrier screening. This approach enabled identification of common, rare and novel variants, significantly improving carrier detection and identifying 35 additional at-risk couples compared with traditional strategies (Shang et al., 2017). Similar benefits were reported in a study of 944 couples using a combined GAP-PCR and NGS-based workflow. The study highlighted the high-throughput capacity and cost effectiveness, which was comparable to their other traditional methods (GAP-PCR and reverse dot blot), while enabling detection of a broader spectrum of common, rare, annotated and novel variants (Zhao et al., 2020). However, as noted, one of the limitations associated with the discovery of an increased number of novel variants is the limited knowledge regarding their pathogenicity and phenotypic manifestation (Zhao et al., 2020).

Targeted NGS has also been explored in the context of newborn screening. A UK pilot study integrating *HBB* sequencing into routine newborn screening demonstrated feasibility and high analytical performance, with 100% analytical sensitivity and 99.96% specificity (van Campen et al., 2019). The major drawback was identified as the associated costs compared to current screening methods, ultimately limiting its use as a first-line test. However, as noted by the authors and others, cost-effectiveness may shift with consolidated testing for other conditions for which NGS-based approaches may also be beneficial (Quarmyne et al., 2025; van Campen et al., 2019). Quarmyne et al. recently discussed the need for a paradigm shift in US newborn screening programs for hemoglobinopathies, advocating universal molecular genetic testing, exemplifying the benefits of NGS-based approaches over those currently used (Quarmyne et al., 2025).

Third-generation (also referred to as long-read) sequencing has also been explored in thalassemia diagnostics (Fan et al., 2023; Peng et al., 2022; Liang et al., 2023). By generating long sequencing reads, this approach may be efficient in the identification of hemoglobinopathy-associated genetic variants, with improved resolution of complex structural variants and homologous regions within globin gene clusters compared with PCR-based methods (Liang et al., 2023). However, long-read sequencing is not yet widely adopted in routine clinical laboratories but may be utilized more widely in the future due to its associated benefits.

### 7.3 Clinical implementation and available commercial assays

In practice, the role of NGS within diagnostic algorithms may vary depending on the clinical setting, laboratory practice, expertise as well as available infrastructure and resources. In regions where a limited number of common variants account for most cases, targeted assays such as GAP-PCR, reverse hybridization methods or MLPA remain efficient first-line approaches. However, in many settings, the underlying variant spectrum is far more heterogeneous, limiting the effectiveness of fixed variant panels as initial testing. NGS is often applied when initial targeted testing is inconclusive, when hematological findings are atypical or when rare or population-specific variants are suspected. In some settings, particularly those with high genetic diversity, NGS may be used as a first-tier method because of its ability to assess multiple genes and variant types simultaneously. Within newborn screening contexts, sequencing approaches are generally used as follow-up or confirmatory testing rather than as a first-line screening tools, although this may evolve as sequencing becomes more accessible and integrated into broader screening panels.

A range of commercial assays is available for thalassemia and sickle cell disease testing, covering both established targeted molecular methods and sequencing-based platforms (Table 2). Targeted assays are available from multiple providers. Examples include reverse hybridization strip assays such as the IVDR-certified α-Globin StripAssay® from ViennaLab Diagnostics, which targets 21 predefined α-globin variants, and several IVDD-certified β-globin assays with region-specific variant panels or modifier loci. Additional targeted assays are available from providers such as MRC Holland and Nuclear Laser Medicine (see Table 2 for further details).

Commercially available NGS-based solutions include, for example, the IVDD-certified THALASSEMIA panel from 4bases and the IVDR-certified Devyser Thalassemia v2 assay from Devyser (Table 2). Published independent studies have reported high sensitivity and broad variant detection capability across diverse populations, with shorter turnaround time relative to sequential targeted testing (Selvatici et al., 2024). As with other NGS approaches, increased detection of variants of uncertain clinical significance remains a limitation (Selvatici et al., 2024). Independent data from Malaysia similarly demonstrated that targeted NGS testing can provide more comprehensive genotyping and facilitate diagnostic workflows, even in resource-limited settings (Zulkeflee et al., 2023).

As with any platform, laboratories evaluating commercial assays should ensure appropriate validation and consider local population genetics, workflow requirements, interpretation processes and clinical practice frameworks when selecting a testing strategy. Guidance on the diagnostic use of NGS is provided by the American College of Medical Genetics and Genomics (ACMG) technical standard (Rehder et al., 2021), together with European recommendations issued by EuroGentest and the European Society of Human Genetics (Matthijs et al., 2016). As the volume and complexity of information generated by NGS increase, challenges related to clinical interpretation, including ambiguous/incidental findings and variants of uncertain significance, remain important considerations, particularly in screening and carrier testing contexts. Professional organizations such as the TIF, EMQN, British Society for Haematology (BSH) and the American Society of Hematology (ASH) continue to play an essential role in establishing consensus recommendations that support standardization of testing, interpretation and reporting, thereby facilitating an appropriate integration of NGS into hemoglobinopathy care.

Collectively, available evidence demonstrates that NGS-based platforms can accommodate the genetic complexity of hemoglobinopathies and provide comprehensive molecular profiles for both diagnostic and screening follow-up applications. At the same time, professional guidelines and commercial assay providers emphasize the continued importance of complementary confirmatory methods, supporting the coexistence of both sequencing-based testing and traditional molecular assays within integrated workflows.

## 8 Discussion and future perspectives

Advances in molecular genetics have substantially expanded the understanding of the genetic basis of thalassemia and sickle cell disease, revealing extensive allelic heterogeneity and the significant contribution of genetic modifiers to clinical variability. The high carrier frequencies, wide geographic distribution and increasing prevalence in traditionally low-incidence regions, driven by population migration and improved survival, underscore the need for robust, scalable and context-appropriate screening and diagnostic strategies. Accurate and timely identification of disease-causing variants and modifiers that influence disease severity and clinical course is central to diagnosis, risk stratification, patient management and genetic counselling. However, these results also require reliable bioinformatic pipelines and careful clinical interpretation to ensure that findings are applied appropriately within healthcare practice.

Progress in sequencing technologies, particularly the evolution and increased availability of NGS-based platforms, now enables a more comprehensive molecular characterization than was previously feasible with conventional, sequential testing workflows. With broad analytical scope, NGS provide an efficient way of decoding the genetic complexity underlying hemoglobinopathies. At the same time, important limitations remain, necessitating guidance and recommendations in the field. Challenges persist with respect to novel and uncertain variant interpretation as well as detection of certain complex structural rearrangements. There is a continued need for complementary confirmatory methods. Practical considerations, including cost, infrastructure, expertise and implementation capabilities across healthcare settings, continue to influence the integration in routine practice.

Looking ahead, continued refinement of sequencing platforms, bioinformatic pipelines and guiding frameworks, together with expanding variant databases and improved understanding of genotype-phenotype correlations, is expected to further strengthen the clinical utility of comprehensive molecular testing in hemoglobinopathies. The integration of advanced sequencing technologies, including NGS, into screening, follow-up and diagnostic workflows represents a key step toward more comprehensive, efficient and personalized hemoglobinopathy care.
